# Evaluation of the effect of the COVID-19 pandemic on mucormycosis studies with bibliometric analysis

**DOI:** 10.1097/MD.0000000000032118

**Published:** 2022-12-02

**Authors:** Tugba Arslan Gulen, Tuba Turunc, Ahmet Riza Sahin, Ebru Oruc, Mehmet Nurullah Kurutkan

**Affiliations:** a Department of Infectious Diseases and Clinical Microbiology, University of Health Sciences Adana City Training and Research Hospital, Adana, Turkey; b Department of Health Management, Faculty of Business, Düzce University, Düzce, Turkey.

**Keywords:** bibliometric analysis, complication, COVID-19, Mucormycosis, research trends, *Rhizopus oryzae*

## Abstract

Coronavirus disease 2019 (COVID-19)-associated mucormycosis (CAM) is a relatively new concept in the literature that emerged during the pandemic. Bibliometric analysis is a type of analysis that uses mathematical and statistical methods to study the formal properties of knowledge areas. This study aimed to reveal the main themes, conceptual structures, and trends of bibliometric studies on mucormycosis in 2 different periods, pre-and during the pandemic.

**Methods:**

This study consisted of 2 periods: pre-COVID-19 and COVID-19. Articles were collected from the Web of Science (WOS) Core Collection database. We provided AND and OR connectors for the keyword query and selected studies based on relevant keywords. Collected data were classified based on their publication date and examined using the R programming language (Version 4.0.3) package Bibliometrix and SciMAT Software.

**Results:**

A total of 1261 articles were investigated, and performance and information structure analyses were conducted. Based on Bradford’s law, the Journal of Fungi was the top-ranked journal in both periods. Cureus and mycoses were placed 2nd and 3rd in the second period. India is the largest contributor. In performance analysis, conceptual structures such as *Rhizopus oryzae*, epidemiology, diagnosis, management, treatment, and outcomes were at the forefront of mucormycosis publications during the COVID-19 period.

**Conclusions:**

Research trends have shifted to the clinical treatment and management of COVID-19. Therefore, pathogenesis, diagnosis, follow-up, and treatment strategies for CAM should be developed in the future.

## 1. Introduction

Mucormycosis is an opportunistic infection caused by saprophytic fungi belonging to the order of Mucorales. The infection may involve the rhinocerebral area, different organs, and systems, such as the central nervous system, lungs, skin, and gastrointestinal system, or may progress with dissemination. Despite being rare, it has high morbidity and mortality rates. Diabetes mellitus (DM) was the most common comorbidity in approximately half of the cases. This issue is frequently observed in immunocompromised hematopoietic stem cell recipients and in cases of solid organ transplant hematological malignancies.^[1]^ The number of mucormycosis cases has increased at a comparatively higher rate than in previous years during the coronavirus disease 2019 (COVID-19) pandemic. The widespread use of high-dose steroids and interleukin antagonists for the treatment of COVID-19 is a risk factor for mucormycosis. Nonetheless, studies have also reported that due to the increased workload in the health system during the pandemic, there has been an increase in mucormycosis during this period as a result of disruptions in the follow-up and treatment of chronic diseases such as DM and chronic kidney disease.^[2–5]^

Bibliometric analysis is a tool that statistically evaluates articles published on a particular subject or field within a specific range of dates. It refers to the analysis of many scientific publications using various statistical technics.^[6]^ The rapidly expanding bibliometric analysis studies offer empirical data on scientific findings. In addition, it enables the comparison of various scientific data of countries, institutions, and researchers, while allowing temporal trend analysis.^[7–9]^ Likewise, the bibliometric analysis presents data summarized in a particular field, and researchers can evaluate new historical and recent data from different perspectives.^[10]^ However, despite several bibliometric studies on infectious diseases in the literature, few comprehensive studies have focused on summarizing and presenting research on mucormycosis, as they also have limited content.^[11,12]^ Moreover, they can make comparisons between general journals publishing the results of large trials and specialty journals, which are leaders in publishing novelties. The network visualization figures make it easier for readers to understand articles, citations, co-citations, and other bibliometric analysis findings by visualizing them.^[13]^ Therefore, we believe there is a remarkable gap in the literature considering the increased number of cases of mucormycosis and high mortality during the pandemic. The course of articles on mucormycosis provides an assessment of worldwide interest, facilitating the evaluation of trends in related research activities over time. Finally, data can raise awareness, and researchers can provide information and support for health policymakers.

This study applied a scientific mapping method to determine the collective focal idea of mucormycosis studies, especially during the COVID-19 period, the development direction of the studies so far, and their influence on scientific research. Finally, the forefront countries and authors were evaluated.

## 2. Materials and Methods

### 2.1. Data source and search strategy

**Figure 1.**
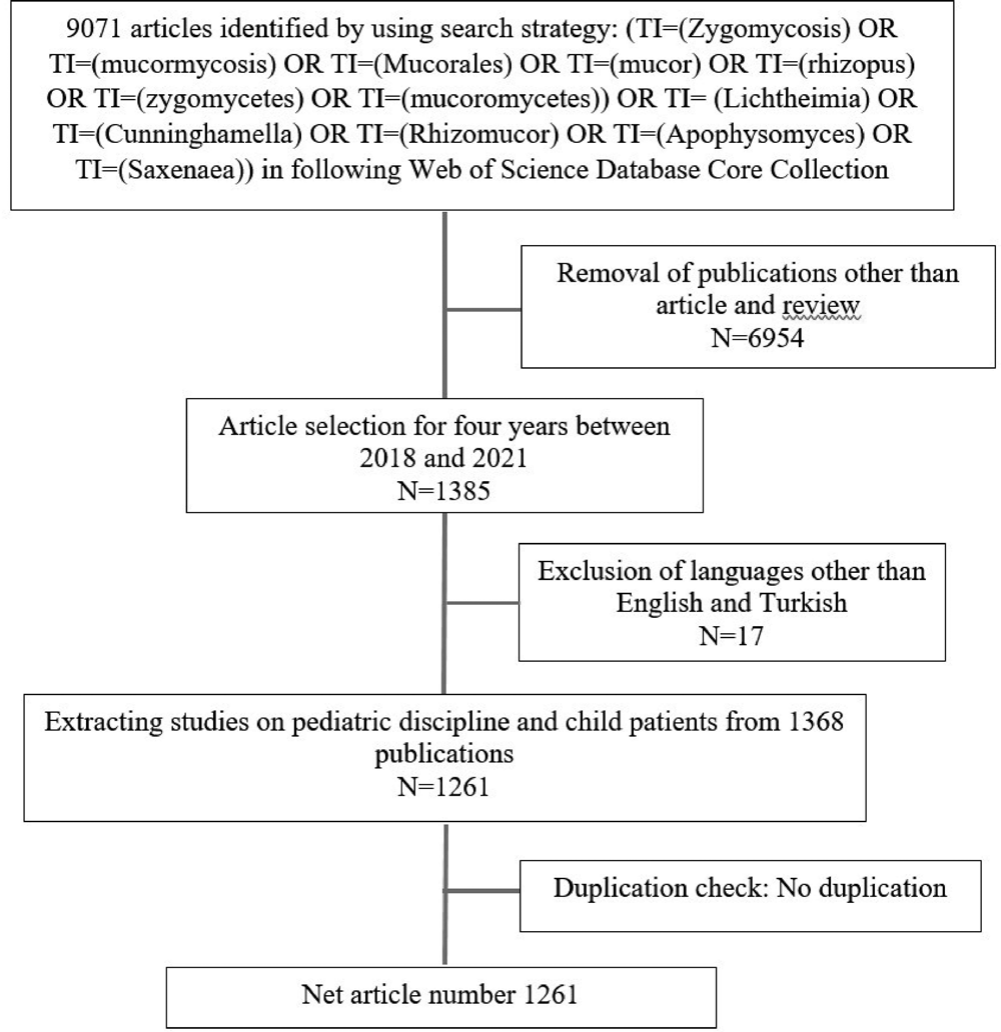
The flow chart.

Two periods, pre-COVID-19 and during-COVID-19, were defined, and the articles published in these 2 periods were analyzed separately. Initially, the pre-COVID-19 period consisted of the period between January 01, 2018, and December 31, 2019, while the during-COVID-19 period covered the period between Jan 01, 2020, and December 18, 2021. All articles in the study dataset were obtained from the Web of Science (WOS) Core Collection. The keyword query was set with AND and OR connectors, and related keywords were searched in the article titles. We defined 1261 articles as the result of the query applied on December 18, 2021. However, studies on pediatric cases were not included in the analysis, and the search strategy is as follows: (TI = (Zygomycosis) OR TI = (mucormycosis) OR TI = (Mucorales) OR TI = (mucor) OR TI = (rhizopus) OR TI = (zygomycetes) OR TI = (mucoromycetes)) OR TI = (Lichtheimia) OR TI = (Cunninghamella) OR TI = (Rhizomucor) OR TI = (Apophysomyces) OR TI = (Saxenaea)) AND ((DT==(“ARTICLE” OR “REVIEW”) AND PY==(“2021) “ OR “2020” OR “2019” OR “2018”)) NOT (TASCA==(“PEDIATRICS”) OR EDN==(“WOS.BSCI” OR “WOS.ISTP” OR “WOS.IC”))) NOTE (TS = CHILD). The articles were then collected by 2 researchers (TAG and MNK) (access date:18.12.2021). Duplicate articles were removed, and a data pool was created. Only the original articles and reviews were obtained from the data pool. At the same time, other types of publications such as book chapters, meeting abstracts, scientific letters, editorial papers, and corrections were disregarded from further analyses. The article titles and abstracts were manually examined, and unrelated articles were removed (Fig. 1).

### 2.2. Data analysis

Raw data were analyzed using Bibliometrix, an R programming language package.^[14]^ The bibliometric analysis of the articles was performed using the R 4.0.3 package program. Units such as the dataset, sources, authors, and documents of the downloaded articles were examined using bibliometric analysis. We also used SciMAT analysis for motor themes and thematic evolution analysis.

Following the framework provided above, the research questions of this study were as follows:

• Can the performance indicators of publications on mucormycosis be examined? To answer this question, annual publication numbers, core journals according to Bradford’s law, h-index values of journals, number of publications per country, and h-index values of authors studying this subject were analyzed.
• Can the knowledge base of publications on mucormycosis and the major themes according to specific periods be determined clearly? Multiple correspondence analysis (MCA), factorial mapping, co-occurrence network, and thematic map analyses were performed to answer this question.

### 2.3. Research ethics

The research was conducted as a bibliometric analysis. All data sources were available on the Internet, and no animal or human subjects were involved. Therefore, permission was not required from the ethics committee.

## 3. Results

The findings are reported under 2 main headings: performance analysis and knowledge base. The performance analysis included the main findings, annual publication numbers, Bradford’s law, impact values of journals and authors (h, g, m index), and number of publications per country, while the knowledge-based analysis consisted of conceptual structure and major theme analysis.

### 3.1. Performance analysis findings

As shown in Table 1, the number of journals in the first and second periods was 321 and 344, respectively. While the rate of citations per article was 8379 in the first period, it was 3658 in the second. In addition, the number of studies with multiple authors was high in both periods, and there were ten articles in the first period and 6 articles in the second period with a single author. Finally, the collaboration index of the authors was above 5 in both periods, which indicates that many researchers worked in collaboration in medical processes.

**Table 1:** Summary information on retrieved mucormycosis publications.

Main information about data			
Timespan	2018–2019	2020–2021	2018–2021
Sources (Journals, Books, etc)	321	344	577
Documents	531	647	1261
Average citations per documents	8.379	3.658	5.462
References	13058	15631	26696
Document types			
Article	485	561	1119
Review	46	86	142
Document contents			
Keywords Plus (ID)	1482	1285	2302
Author’s Keywords (DE)	1379	1691	2863
Authors			
Authors	2647	3576	6132
Authors of multi-authored documents	2637	3571	6116
Authors collaboration			
Single-authored documents	10	6	16
Documents per author	0.201	0.181	0.206
Authors per document	4.98	5.53	4.86
Co-Authors per documents	6.11	6.59	6.34
Collaboration index	5.06	5.56	4.91

**Figure 2.**
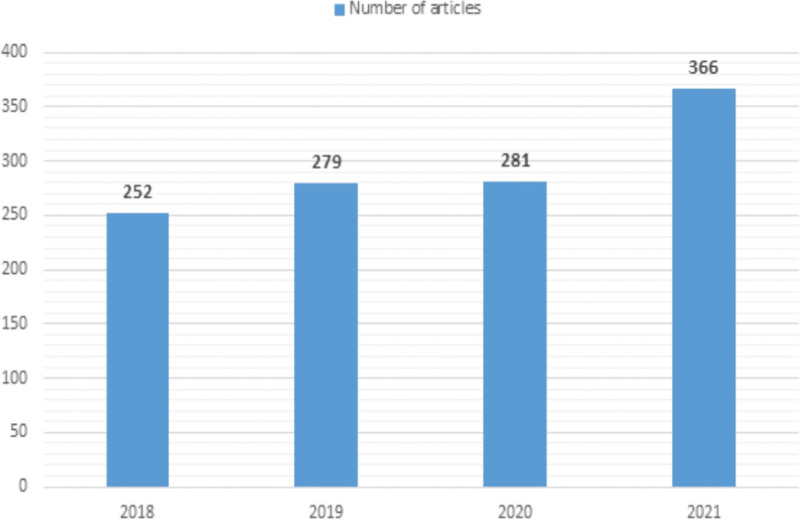
Annual number of publications (Annual Growth Rate 13.25%).

Additionally, we observed an increasing trend in the number of publications per year. Initially, the overall number of articles was 252, which soared to 366 in 2021 (Fig. 2).

According to Bradford’s law, studies in a particular field are usually published from certain sources. Thus, sorted in descending order according to the number of publications, the sources were classified into 3 groups, each covering one-third of the publications. The first group consists of core resources.^[15]^ Within this framework, the Journal of Fungi, Medical Mycology Case Reports, and Journal de Mycologie Medicale were among the top 3 in the first period. Nonetheless, in the second period, while there was no change in the first place, the journal Cureus ranked second and Mycoses ranked third (Table 2).

**Table 2:** Core journals that included in zone 1 of Bradford’s law.

	2018–2019			2020–2021		
	Source	R	F	Source	R	F
1	Journal of Fungi	1	15	Journal of Fungi	1	22
2	Medical Mycology Case Reports	2	15	Cureus	2	20
3	Journal de Mycologie Medicale	3	9	Mycoses	3	17
4	Mycopathologia	4	9	Medical Mycology Case Reports	4	13
5	Mycoses	5	9	IDcases	5	12
6	Medical Mycology	6	8	International Journal of Biological Macromolecules	6	11
7	Antimicrobial Agents and Chemotherapy	7	7	LWT-Food Science and Technology	7	8
8	Scientific Reports	8	7	Mycopathologia	8	8
9	Frontiers in Microbiology	9	6	BMJ Case Reports	9	7
10	IDcases	10	6	Diabetes & Metabolic Syndrome-Clinical Research & Reviews	10	7
11	International Journal of Biological Macromolecules	11	6	Journal De Mycologie Medicale	11	7
12	Cureus	12	5	Frontiers in Microbiology	12	6
13	Indian Journal of Otolaryngology and Head & Neck Surgery	13	5	International Journal of Surgery Case Reports	13	6
14	Journal of Clinical and Diagnostic Research	14	5	Journal of Clinical and Diagnostic Research	14	6
15	MBIO	15	5	Medical Science	15	6
16	Phytotaxa	16	5	Frontiers in Medicine	16	5
17	Applied Biochemistry and Biotechnology	17	4	Indian Journal of Critical Care Medicine	17	5
18	Case Reports in Infectious Diseases	18	4	Indian Journal of Ophthalmology	18	5
19	Food Chemistry	19	4	Medical Mycology	19	5
20	Journal of Antimicrobial Chemotherapy	20	4	Microorganisms	20	5
21	Journal of Clinical Microbiology	21	4	Molecules	21	5
22	Process Biochemistry	22	4	Process Biochemistry	22	5
23	Transplant Infectious Disease	23	4	Applied Biochemistry and Biotechnology	23	4
24	AMB Express	24	3	Biocatalysis and Agricultural Biotechnology	24	4
25	Clinical Case Reports	25	3	Bioresource Technology	25	4
26	Environmental Science and Pollution Research	26	3	BMC Infectious Diseases	26	4
27	Fuel	27	3	Case Reports in Infectious Diseases	27	4
28	Fungal Genetics and Biology	28	3	Catalysts	28	4
29	International Journal of Clinical and Experimental Medicine	29	3			
30	International Journal of Molecular Sciences	30	3			
31	Journal of Agricultural and Food Chemistry	31	3			
32	Journal of Thoracic Disease	32	3			

F = frequency, R = rank.

The h, g, and m index values of the sources and numerical data regarding the total number of citations are listed in Table 3. The citation number of the Hirsch h index predicts future success better than indicators such as the number of citations, number of articles, and average citations per article.^[16]^ The journal with the highest h-index is the Journal of Fungi, with a value of 10 in the first and 7 in the second period. This index shows that ten articles published in the journal received more than ten citations. Additionally, it has the feature of having the highest g index and total number of citations.

**Table 3:** Impact values analysis of journals.

2018–2019 Period					
Journals	h	g	m	TC	NP
Journal of Fungi	10	15	2.5	401	15
Medical Mycology	7	8	1.75	269	8
International Journal of Biological Macromolecules	6	6	1.5	96	6
Mycoses	6	8	1.5	77	9
Scientific Reports	6	7	1.5	69	7
Frontiers in Microbiology	5	6	1.25	76	6
MBIO	5	5	1.25	85	5
Antimicrobial Agents and Chemotherapy	4	6	1	38	6
Journal of Antimicrobial Chemotherapy	4	4	1	64	4
Journal of Clinical Microbiology	4	4	1	84	4
Process Biochemistry	4	4	1	22	4
AMB Express	3	3	0.75	24	3
Applied Biochemistry and Biotechnology	3	4	0.75	66	4
Food Chemistry	3	4	0.75	108	4
Fuel	3	3	0.75	25	3
Fungal Genetics and Biology	3	3	1	32	3
IDcases	3	3	0.75	15	4
Indian Journal of Otolaryngology and Head & Neck Surgery	3	4	0.75	21	5
International Journal of Molecular Sciences	3	3	0.75	39	3
Journal de Mycologie Medicale	3	6	0.75	40	7
2020–2021 Period					
JOURNALS	h	g	m	TC	NP
Journal of Fungi	7	14	3.5	212	16
Mycoses	6	10	3	131	16
Indian Journal of Ophthalmology	5	5	5	145	5
Mycopathologia	4	6	2	110	6
Biocatalysis and Agricultural Biotechnology	3	3	1.5	20	3
Bioresource Technology	3	4	1.5	18	4
Cureus	3	9	1.5	159	9
Diabetes & Metabolic Syndrome-Clinical Research & Reviews	3	5	3	102	5
Food Chemistry	3	3	1.5	36	3
Genes	3	3	1.5	27	3
International Journal of Biological Macromolecules	3	4	1.5	27	8
Medical Mycology Case Reports	3	7	1.5	50	7
Molecules	3	3	1.5	13	5
Postharvest Biology and Technology	3	4	1.5	32	4
Process Biochemistry	3	4	1.5	27	4
Applied Biochemistry and Biotechnology	2	2	1	6	3
Applied Biological Chemistry	2	2	1	7	2
Biocatalysis and Biotransformation	2	3	1	9	3
Biointerface Research in Applied Chemistry	2	2	1	8	3
Bioprocess and Biosystems Engineering	2	2	1	7	2

h = Hirsch index or Hirsch number, g = g-index is an author or journal-level metric. The g-index is an alternative for the older h-index, m = The m-index is defined as h/n, where h is the h-index and n is the number of years since the first published paper of the journal, TC = total citation, NP = number of production.

The m index is defined by Hirsch and is obtained by dividing the h index by the number of years the academic is active.^[17]^ Therefore, the indices show that the most influential journals in the first period were the Journal of Fungi, Medical Mycology, International Journal of Biological Macromolecules, Mycoses, and Scientific Reports. The Journal of Fungi, Mycoses, and the Journal of Ophthalmology were in the top 3 in the top 3. Thus, we can infer that the most prominent studies were published in these journals. Thus, these journals with the highest ranks must be prioritized in the decision-making and policy-making processes and future related studies.

When Table 4 is examined, it was found that the countries that contributed the most to the literature on the subject in the first period were the USA, China, Germany, France, and India, while India took first place in the COVID-19 period and the entire period.

**Table 4:** Publications per countries.

	2018–2019		2020–2021		2018–2021	
	Country	F	Country	F	Country	F
1	USA	282	India	578	India	687
2	China	233	China	281	USA	544
3	Germany	138	USA	262	China	514
4	France	117	Iran	111	Germany	220
5	India	109	France	84	France	201
6	Brazil	92	Germany	82	Iran	192
7	Iran	81	Brazil	80	Brazil	172
8	Japan	78	Egypt	72	Japan	140
9	Mexico	62	Japan	62	Mexico	119
10	Spain	54	Saudi Arabia	61	Spain	109
11	UK	36	South Korea	59	Egypt	90
12	Australia	33	Mexico	57	South Korea	90
13	South Korea	31	Spain	55	Saudi Arabia	80
14	Thailand	29	Turkey	45	Turkey	71
15	Canada	28	Netherlands	34	UK	59
16	Poland	27	Italy	31	Australia	54
17	Turkey	26	Malaysia	30	Thailand	53
18	Greece	25	Thailand	24	Italy	52
19	Austria	21	UK	23	Malaysia	48
20	Italy	21	Austria	22	Netherlands	47
21	Saudi Arabia	19	Australia	21	Canada	45
22	Egypt	18	Portugal	21	Austria	43
23	Malaysia	18	Czech Republic	19	Poland	43
24	Pakistan	17	Canada	17	Pakistan	34
25	Switzerland	17	Denmark	17	Greece	32
26	Belgium	16	Nepal	17	Portugal	27
27	Israel	15	Nigeria	17	Switzerland	27
28	Indonesia	13	Pakistan	17	Israel	25
29	Netherlands	13	Poland	16	Argentina	24
30	Argentina	12	Peru	15	Czech Republic	24
31	Colombia	10	Qatar	14	Indonesia	21
32	Ireland	10	Hungary	13	Nigeria	21
33	Tunisia	9	Argentina	12	Denmark	20
34	Ukraine	8	Tunisia	11	Tunisia	20
35	Romania	7	Israel	10	Hungary	19
36	Croatia	6	Singapore	10	Belgium	17
37	Hungary	6	Switzerland	10	Nepal	17
38	Portugal	6	Indonesia	8	Colombia	15
39	South Africa	6	Greece	7	Peru	15
40	Czech Republic	5	South Africa	7	Qatar	15
41	Lebanon	5	Sweden	7	Ireland	14
42	Russia	5	Romania	6	Romania	13
43	Bahrain	4	Russia	6	South Africa	13
44	Nigeria	4	Bahrain	5	Singapore	12
45	Denmark	3	Colombia	5	Russia	11
46	Kuwait	3	Bulgaria	4	Ukraine	11
47	Serbia	3	Ireland	4	Bahrain	9
48	Sri Lanka	3	Chile	3	Sweden	8
49	Albania	2	Iraq	3	Croatia	6
50	Algeria	2	Kyrgyzstan	3	Serbia	6
51	Iraq	2	Mauritius	3	Iraq	5
52	New Zealand	2	Serbia	3	Lebanon	5
53	Singapore	2	Slovakia	3	Slovakia	5
54	Slovakia	2	Ukraine	3	Bulgaria	4
55	Cyprus	1	Vietnam	3	Sri Lanka	4
56	Morocco	1	Ethiopia	2	Chile	3
57	Oman	1	Kazakhstan	2	Kuwait	3
58	Paraguay	1	Morocco	2	Kyrgyzstan	3
59	Qatar	1	Norway	2	Mauritius	3
60	Sweden	1	Oman	2	Morocco	3
61			Afghanistan	1	Oman	3
62			Bangladesh	1	Vietnam	3
63			Belgium	1	Albania	2
64			Bolivia	1	Algeria	2
65			Eritrea	1	Ethiopia	2
66			Sri Lanka	1	Kazakhstan	2
67			Sudan	1	New Zealand	2
68			Yemen	1	Norway	2
69					Afghanistan	1
70					Bangladesh	1

F = frequency.

The authors’ h, g, and m index values and total number of citations are provided in Table 5. While the author with the highest h-index was Garre V, with a 9 h-index in the first period, Chakrabarti A had a 7 h-index in the second period. The authors in the first place were also in the first place in the g-index. In the second period, Shah S had the highest m-index. Despite the relatively short academic life of the related author, he was quite productive.

**Table 5:** Authors with the highest author impact analysis index value.

2018–2019 period						2020–2021 period					
Author	h	g	m	TC	NP	Author	h	g	m	TC	NP
Garre V	9	12	2.250	179	12	Chakrabarti A	7	11	3.5	224	11
Cornely OA	8	8	2.000	375	8	Garre V	6	8	3	73	12
Heitman J	7	7	1.750	138	7	Muthu V	6	7	3	182	7
Kontoyiannis DP	7	8	1.750	150	8	Agarwal R	5	7	2.5	165	7
Song YD	7	9	1.750	102	12	Navarro E	4	6	2	46	7
Dannaoui E	5	6	1.250	293	6	Perez-Arques C	4	5	2	44	5
Hoenigl M	5	5	1.250	349	5	Rudramurthy SM	4	7	2	63	7
Ibrahim AS	5	5	1.250	358	5	Shah S	4	4	4	91	4
Navarro-Mendoza MI	5	5	1.250	93	5	Alkhazraji S	3	3	1.5	36	3
Nicolas FE	5	5	1.250	93	5	Alqarihi A	3	3	1.5	36	3
Perez-Arques C	5	6	1.250	96	6	Cornely OA	3	4	1.5	29	4
Voigt K	5	5	1.250	84	5	Filler SG	3	3	1.5	36	3
Xu L	5	6	1.250	99	6	Gebremariam T	3	3	1.5	36	3
Alastruey-Izquierdo A	4	4	1.000	297	4	Gupta S	3	3	1.5	40	3
Attan N	4	4	1.000	80	4	Hussain SA	3	3	1.5	18	3
Bento HBS	4	4	1.000	60	4	Ibrahim AS	3	3	1.5	36	3
Chakrabarti A	4	4	1.333	463	4	Kaur H	3	3	1,5	123	3
Chen KS	4	6	1.000	60	6	Kumar S	3	4	3	36	4
De Castro HF	4	4	1.000	60	4	Kurzai O	3	3	1.5	30	3
Gamarra S	4	4	1.000	26	4	Navarro-Mendoza MI	3	4	1.5	35	4

h = Hirsch index or Hirsch number, g = g-index is an author or journal-level metric. The g-index is an alternative for the older h-index, m = The m-index is defined as h/n, where h is the h-index and n is the number of years since the first published paper of the scientist, TC = total citation, NP = number of production.

Table 6 shows the top 10 most cited articles in both periods. Cornely et al’s article titled “Global Guideline For the Diagnosis and Management Of Mucormycosis: An Initiative of The European Confederation Of Medical Mycology in Cooperation with the Mycoses Study Group Education And Research Consortium” ranks first with 248 citations in the first period. The second-ranked article is a meta-analysis study that includes epidemiology and clinical manifestations and has 153 citations. During the pandemic period, the case report of Mehta and Pandey titled “Rhino-Orbital Mucormycosis Associated with COVID-19,” which has 110 citations, is in the first place. Sing et al’s work titled “Mucormycosis in COVID-19: A Systematic Review of Cases Reported Worldwide and in India” ranks second.

**Table 6:** The 10 most cited articles in 2 periods.

**2018–2019**				**2020–2021**			
Authors	Title	Journal	C	Authors	Title	Journal	C
Cornely et al (2019)	GLOBAL GUIDELINE FOR THE DIAGNOSIS AND MANAGEMENT OF MUCORMYCOSIS: AN INITIATIVE OF THE EUROPEAN CONFEDERATION OF MEDICAL MYCOLOGY IN COOPERATION WITH THE MYCOSES STUDY GROUP EDUCATION AND RESEARCH CONSORTIUM	JOURNAL OF FUNGI	248	Mehta & Pandey (2020)	RHINO-ORBITAL MUCORMYCOSIS ASSOCIATED WITH COVID-19	CUREUS	110
Jeong et al (2019)	THE EPIDEMIOLOGY AND CLINICAL MANIFESTATIONS OF MUCORMYCOSIS: A SYSTEMATIC REVIEW AND META-ANALYSIS OF CASE REPORTS	LANCET INFECTIOUS DISEASES	153	Singh et al (2021)	MUCORMYCOSIS IN COVID-19: A SYSTEMATIC REVIEW OF CASES REPORTED WORLDWIDE AND IN INDIA	DIABETES & METABOLIC SYNDROME-CLINICAL RESEARCH & REVIEWS	90
Prakash & Chakrabarti (2019)	GLOBAL EPIDEMIOLOGY OF MUCORMYCOSIS	CLINICAL MICROBIOLOGY AND INFECTION	144	Werthman-Ehrenreich et al (2021)	MUCORMYCOSIS WITH ORBITAL COMPARTMENT SYNDROME IN A PATIENT WITH COVID-19	AMERICAN JOURNAL OF EMERGENCY MEDICINE	87
Skiada et al (2018)	CHALLENGES IN THE DIAGNOSIS AND TREATMENT OF MUCORMYCOSIS	JOURNAL OF FUNGI	99	Garg et al (2021)	CORONAVIRUS DISEASE (COVID-19) ASSOCIATED MUCORMYCOSIS (CAM): CASE REPORT AND SYSTEMATIC REVIEW OF LITERATURE	MYCOPATHOLOGIA	81
Prakash et. al (2019)	A PROSPECTIVE MULTICENTER STUDY ON MUCORMYCOSIS IN INDIA: EPIDEMIOLOGY, DIAGNOSIS, AND TREATMENT	MEDICAL MYCOLOGY	56	Sen et al (2021)	MUCOR IN A VIRAL LAND: A TALE OF TWO PATHOGENS	INDIAN JOURNAL OF OPHTHALMOLOGY	74
De Oliveira et. al (2018)	EFFECT OF THE PRESENCE OF SURFACTANTS AND IMMOBILIZATION CONDITIONS ON CATALYSTS’ PROPERTIES OF RHIZOMUCOR MIEHEI LIPASE ONTO CHITOSAN	MEDICAL MYCOLOGY	44	Mekonnen et al (2021)	ACUTE INVASIVE RHINO-ORBITAL MUCORMYCOSIS IN A PATIENT WITH COVID-19-ASSOCIATED ACUTE RESPIRATORY DISTRESS SYNDROME	OPHTHALMIC PLASTIC AND RECONSTRUCTIVE SURGERY	64
Sun et al (2018)	A NOVEL ASPARTIC PROTEASE FROM RHIZOMUCOR MIEHEI EXPRESSED IN PICHIA PASTORIS AND ITS APPLICATION ON MEAT TENDERIZATION AND PREPARATION OF TURTLE PEPTIDES	APPLIED BIOCHEMISTRY AND BIOTECHNOLOGY	42	John et al (2021)	WHEN UNCONTROLLED DIABETES MELLITUS AND SEVERE COVID-19 CONVERGE: THE PERFECT STORM FOR MUCORMYCOSIS	JOURNAL OF FUNGI	63
Adnan et al (2018)	X-SHAPED ZIF-8 FOR IMMOBILIZATION RHIZOMUCOR MIEHEI LIPASE VIA ENCAPSULATION AND ITS APPLICATION TOWARD BIODIESEL PRODUCTION	FOOD CHEMISTRY	42	Skiada et al (2020)	EPIDEMIOLOGY AND DIAGNOSIS OF MUCORMYCOSIS: AN UPDATE	JOURNAL OF FUNGI	56
Kong et al (2019)	ANTIFUNGAL EFFECTS OF THYMOL AND SALICYLIC ACID ON CELL MEMBRANE AND MITOCHONDRIA OF RHIZOPUS STOLONIFER AND THEIR APPLICATION IN POSTHARVEST PRESERVATION OF TOMATOES	CATALYSTS	41	Pasero et al (2021)	A CHALLENGING COMPLICATION FOLLOWING SARS-COV-2 INFECTION: A CASE OF PULMONARY MUCORMYCOSIS	INFECTION	43
Sipsas et al (2018)	THERAPY OF MUCORMYCOSIS	FOOD CHEMISTRY	40	Do Monte et al (2020)	RARE AND FATAL GASTROINTESTINAL MUCORMYCOSIS (ZYGOMYCOSIS) IN COVID-19 PATIENT: A CASE REPORT	CLINICAL ENDOSCOPY	40

C = citation.

### 3.2. Conceptual structure analysis

#### 3.2..1. Multiple correspondence analysis (Keyword plus analysis).

**Figure 3.**
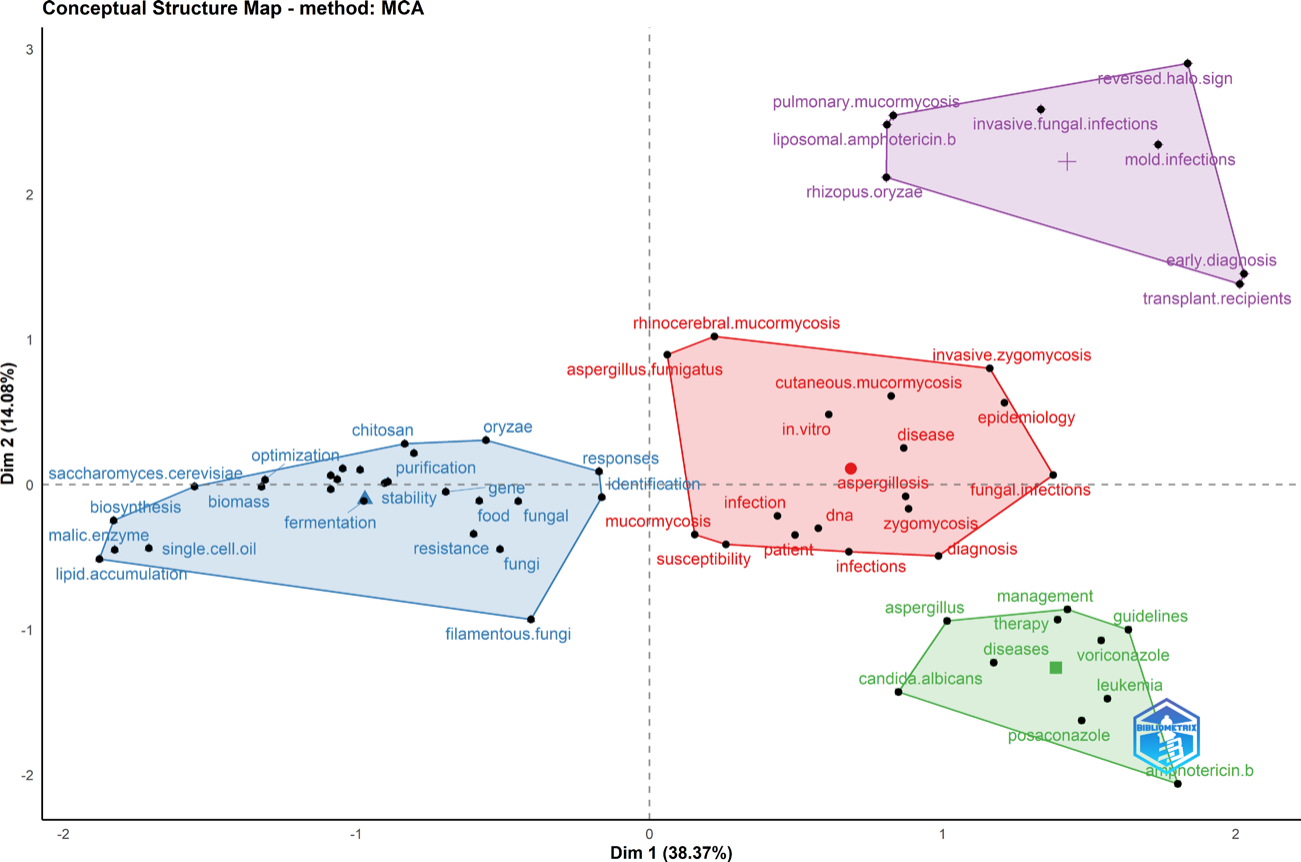
Multiple correspondence analyses of publications.

According to MCA analysis, mucormycosis-associated clusters were grouped under the 4 main cluster titles. Each cluster is represented by a color (red, light blue, light green, or purple). The cluster names and main themes were determined based on the sub-words of the clusters. Consequently, the volume and number of references are directly proportional. Accordingly, the red cluster is more dominant and central, and the red cluster contains the sub-words of the sources involving baseline and main information on managing invasive fungal infections such as epidemiology, diagnosis, susceptibility, and treatment. The cluster also included 2 words in the clinical classification of mucormycosis (rhinocerebral and cutaneous mucormycosis). In contrast, blue is the second most important cluster containing more specific subwords related to microbiological diagnosis, identification, molecular properties, and resistance. Moreover, the purple-colored cluster consisted of keywords associated with COVID-19-associated mucormycosis (CAM), which is dynamically active in recent literature, such as mold infections and liposomal amphotericin B. Pulmonary mucormycosis and *Rhizopus oryzae*, which have an increased incidence of COVID-19, are also sub-words in this cluster (Fig. 3). The green cluster contains general information regarding invasive fungal infections rather than mucormycosis.

#### 3.2..2. Factoriel map analyses.

**Figure 4.**
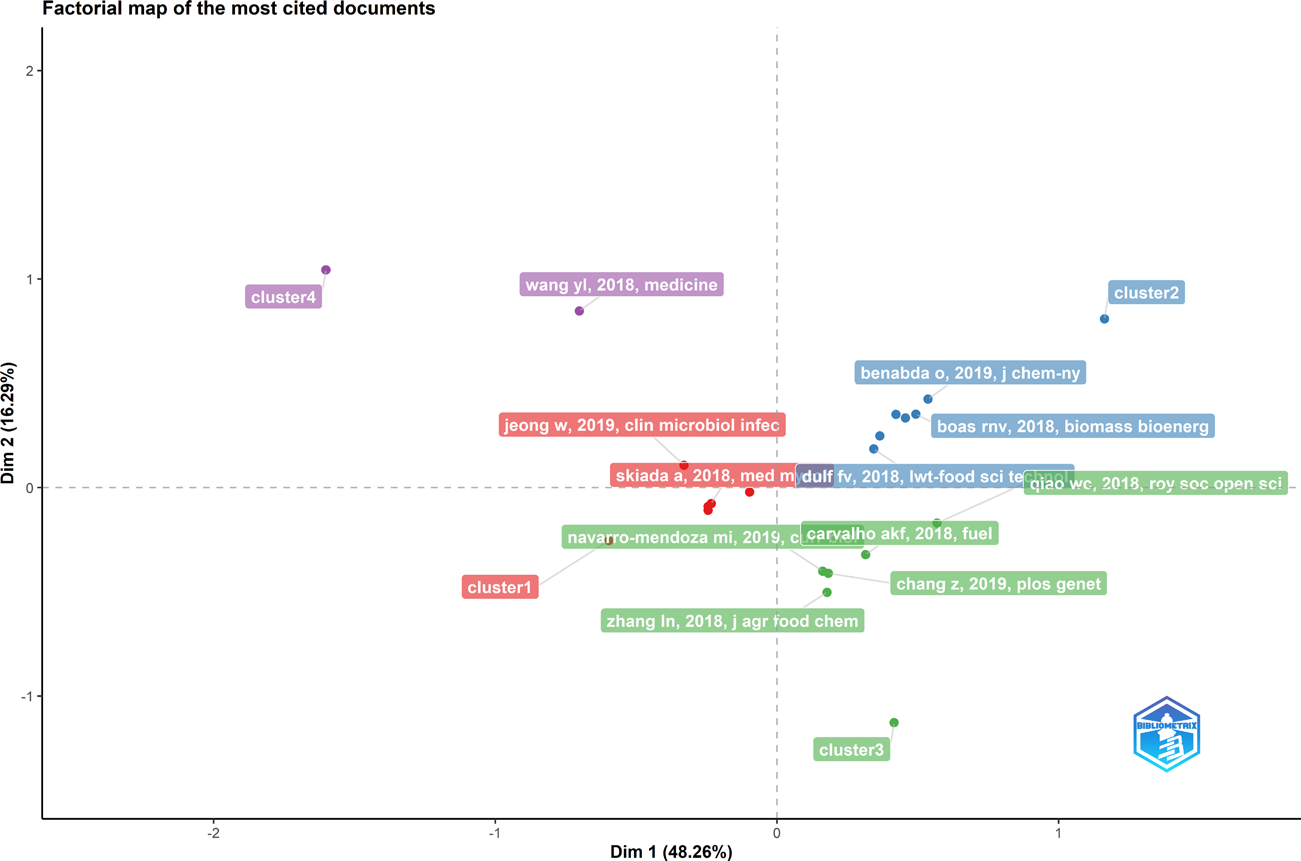
Factorial mapping of the most cited sources (2018–2019).

**Figure 5.**
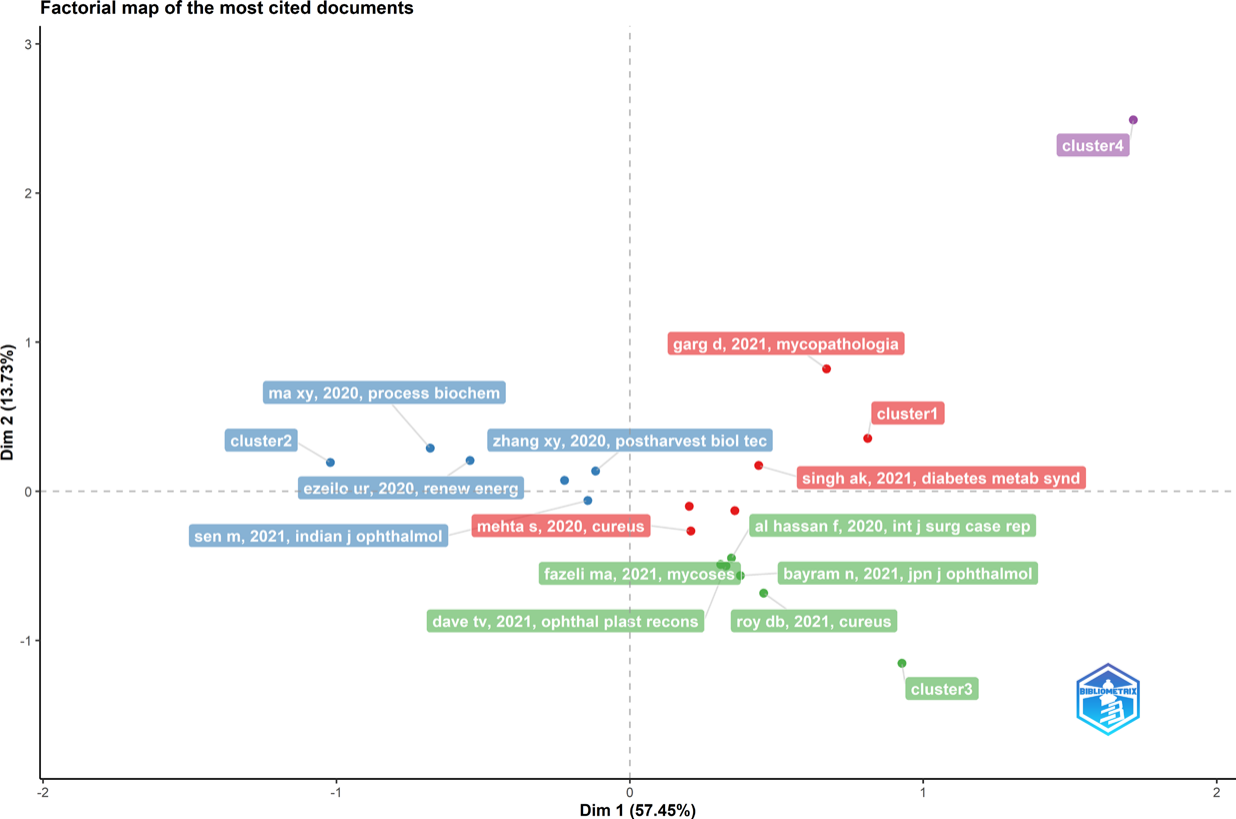
Factorial mapping of the most cited sources (2020–2021).

In Figures 4 and 5, the factorial map of the link between the topic and articles presents the documents associated with the highest total contribution for each period. The total contribution measures the weight of each document in the data summarized by 2 axes, and each color represents a cluster to which each document belongs. The works in the red cluster are the most contributing. Figure 4 illustrates the most-cited studies in each cluster during the first period. The publications titled “Epidemiology and Diagnosis of Mucormycosis: An Update” by Skiada et al^[18]^ and “The epidemiology and clinical manifestations of mucormycosis: a systematic review and meta-analysis of case reports” by Jeong et al^[19]^ were the most cited studies during the first period. Mehta’s case report of rhinocerebral mucormycosis, Garg’s case report of pulmonary mucormycosis associated with COVID-19, and Singh’s study analyzing global CAM case reports were the most cited reports during the pandemic period^[20,21]^ (Fig. 5).

#### 3.2..3. Co-occurrence network analysis.

**Figure 6.**
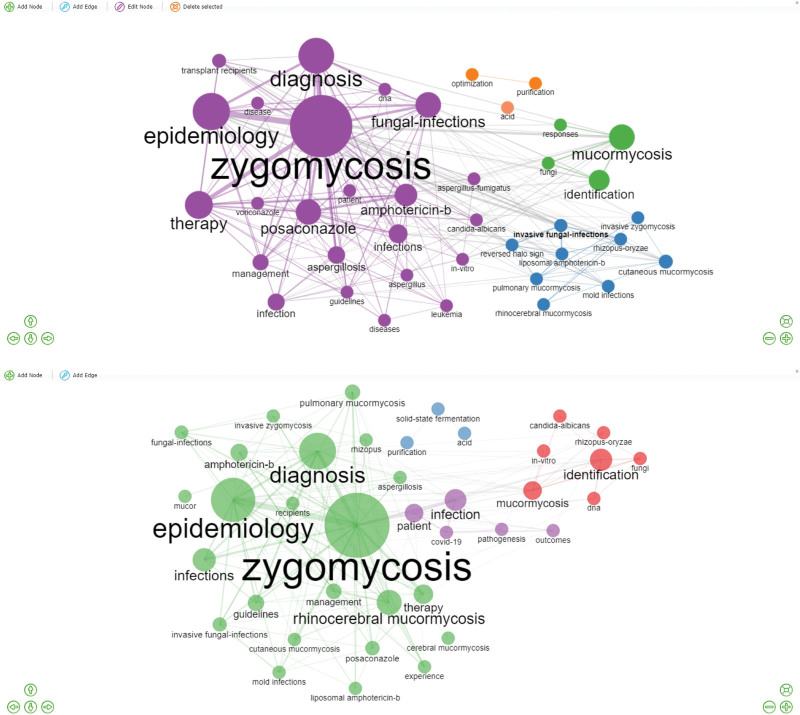
Co-occurrence network analysis (2018–2019 vs 2020–2021).

Similarly, in the co-occurrence network analysis, each color represents a cluster, whereas the size of the circles indicates the number of citations. Four clusters were detected in the first period and 5 clusters in the second period. Therefore, the largest cluster was the purple cluster in the first period, consisting of sub-words such as zygomycosis, epidemiology, diagnosis, therapy, posaconazole, and amphotericin B, which were included in the general information section of all relevant studies. In the second period, the green cluster was the largest and central theme with keywords similar to the largest cluster of the first period, as well as sub-words such as rhinocerebral, pulmonary, cutaneous, and cerebral mucormycosis in the clinical classification of mucormycosis. Remarkably, a new theme has also emerged in the second period, in which COVID-19 is located within the same network where sub-words such as pathogenesis, outcomes, patients, and infection are central topics. In the second period, new sub-words related to diagnosis, such as identification, in vitro, *R oryzae*, and DNA in the red cluster were among the main findings (Fig. 6).

#### 3.2..4. Thematic map analysis.

**Figure 7.**
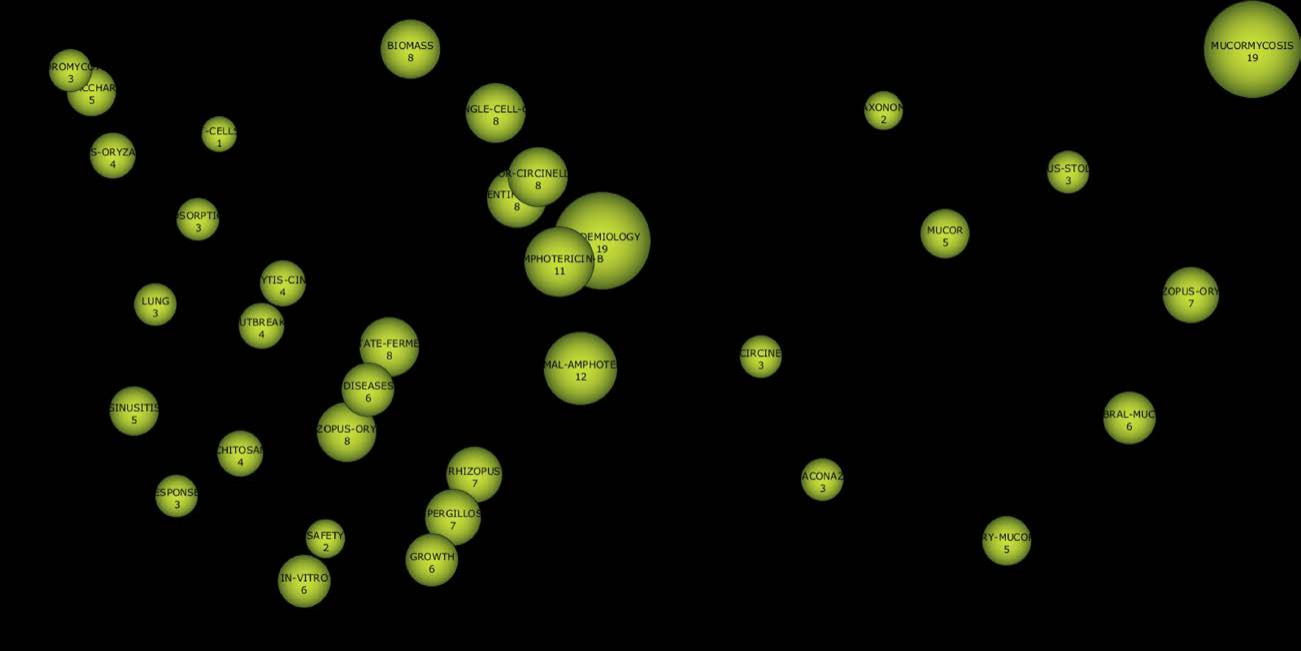
Thematic evolution analysis (Author keyword analysis) (2018–2019 vs 2020–2021).

The developed and most studied themes were visualized in the Q1 major theme area (upper-right area). Six concepts related to cellular properties, identification, epidemiology, and treatment were included in this area in the first period. In the second period, mucormycosis and *R oryzae* were the main concepts of the motor theme. Basic and transformational themes (Q2) (lower right area) include themes that have the highest relations with other external themes but have not developed much in themselves. Although the themes in this field are thought to be related to the research field, they are still not fully developed. When both periods were compared, the concept of *R oryzae*, which was in the Q2 theme in the first period, switched to the Q1 motor theme in the second period. A new cluster emerged in the Q2 theme in the second period, including rhinocerebral mucormycosis and pulmonary mucormycosis. The number of publications on these concepts should be increased and a large number of citations should be made. Emerging or descending themes (Q3) (lower left area) are weak or marginally advanced in the relevant field. During the second period, there was no dominant concept in this field. Niche themes (Q4) (upper left area), on the other hand, are highly developed themes in themselves, although they do not have many relationships with other external themes in the relevant field. These themes are considered well developed in the relevant field and only of marginal importance for the field. In the second period, there were no dominant concepts in the niche (Q4) theme (Fig. 7). As CAM is a new clinical definition, it is expected that the dominant concepts will not be included in themes Q3 and Q4.

## 4. Discussion

Our study aimed to systematize these research areas and reveal specific research themes based on studies published on mucormycosis. To the best of our knowledge, few bibliometric studies have examined mucormycosis. Gupta et al, for instance, provided a surface evaluation by reviewing studies of the 1998 to 2021 period.^[22]^ Likewise, Sharma and Dubey included early publications of post-COVID mucormycosis included in the WHO (World Health Organization) database, yet only 51 articles were analyzed.^[11]^ Mallikarjun et al also analyzed post-COVID mucormycosis studies and evaluated 154 documents.^[12]^ Nonetheless, the time interval in these studies was limited; consequently, the number of studies analyzed was modest. However, we included a broader time interval in the study and evaluated more publications comparing the pre-COVID-19 and during-COVID-19 periods. Thus, this is the first study in the literature to compare both periods.

Our bibliometric analysis showed an overall increase in research articles on mucormycosis during the COVID-19 period. The severe course of COVID-19 is due to hyperactivation of the immune system. As there is no proven and specific treatment, glucocorticoids and immunomodulatory agents have been used frequently, especially in hypoxic patients, since the beginning of the pandemic. However, their use predisposes patients to secondary bacterial and fungal infections. The increase in new DM diagnoses in patients with impaired glucose tolerance, especially those with difficulties in hospital admission due to the pandemic and glucocorticoid treatments, permitted opportunistic infections. Recently, the definition of CAM has been added to the literature, and multifactorial factors such as immune dysregulation of the virus, endothelial dysfunction, immunosuppressive treatments used, uncontrolled DM, and invasive interventions applied to the patient are considered significant risk factors.^[20]^

Regarding contributions by countries, India significantly impacted the literature on the subject during COVID-19, and the author was Chakrabarti. In the second wave of the pandemic, CAM cases have been intensively reported in India since early.^[20,21,23,24]^ Various factors may have contributed to this situation, such as the lack of compliance with the measures due to population density during the pandemic, the severe increase in the number of COVID-19 patients, and the failure to follow-up patients with risk factors for mucormycosis due to the heavy burden on the health system. In addition, India is one of the countries with a high prevalence of DM in the adult age group.^[25]^ A multicenter national study showed that most patients diagnosed with mucormycosis had uncontrolled DM and diabetic ketoacidosis.^[26]^ Muthu et al found a higher proportion of patients with diabetes, while in other countries, they constituted the majority of patients with hematological malignancies and organ transplant recipients.^[27]^

As a result of evaluating the 2 periods in terms of the number of citations, we observed that although the annual number of publications was highest in the second period, the rate of citations per article was lower. This may be because CAM is a newly emerging concept, and articles in the first period were cited in the second period. The Journal of Fungi ranks first in both Bradford’s Law Zone 1 and impact value analysis. Especially in the second period, Cureus, Mycoses, and the Journal of Ophthalmology are also in the top ranks, and these journals can be considered in the selection of journals for future related studies. *R oryzae* is the most frequently isolated agent worldwide,^[28]^ and MCA and thematic map analysis revealed that it was also the predominant agent during the COVID-19 period.

One of the limitations of our study was the use of many terms and words to identify and describe mucormycosis articles. New conceptualizations and denominations emerge over time, and accepting and using these words often requires time. For instance, given the nature of the research, different key terms may reveal different articles that may affect the research results. Future studies should explore the issue of mucormycosis using various bibliometric analyses. The WoS database is widely viewed as having higher trustworthiness than other databases like PubMed and Scopus because it exclusively indexes the journals in the Science Citation Index Expanded and Emerging Science Citation Index.^[29,30]^ As a result, only the WoS database was used in this study and not others like PubMed and Scopus. Finally, we suggest that the intellectual structure of the field can be studied using the techniques used in cluster analysis. Different clusters may emerge based on the requirements of the era.

## 5. Conclusion

In this bibliometric analysis, mucormycosis publications conducted before and during the COVID-19 pandemic were analyzed objectively and comprehensively. While the USA was the country that contributed the most before COVID-19, India overtook it during COVID-19. Moreover, we showed that during the COVID-19 period, in which *R oryzae* was prominent, diagnostic methods such as molecular tests other than microbiological methods were used. Meanwhile, pulmonary and rhinocerebral involvement was highly prevalent, which promoted these concepts. Our results will help to better understand future research trends in mucormycosis. Finally, we believe that new strategies will be developed in the upcoming years, especially for identifying and preventing risk factors for CAM and the follow-up, treatment, and management of these patients. When our analysis results were evaluated, it was observed that conceptual structures such as epidemiology, diagnosis, management, treatment, and results were at the forefront of mucormycosis publications during the COVID-19 period. Studies on pathogenesis, molecular methods, and biomarkers are scarce, so it would be wise to plan research in these areas.

## Author contributions

**Conceptualization:** Tuba Turunc, Ahmet Riza Sahin.

**Data curation**: Tugba Arslan Gulen, Mehmet Nurullah Kurutkan.

**Formal analysis:** Ebru Oruc, Mehmet Nurullah Kurutkan.

**Funding acquisition:** Tugba Arslan Gulen, Ebru Oruc.

**Investigation:** Tuba Turunc, Ahmet Riza Sahin.

**Methodology:** Tugba Arslan Gulen, Mehmet Nurullah Kurutkan.

**Software:** Tugba Arslan Gulen, Tuba Turunc, Mehmet Nurullah Kurutkan.

**Validation:** Ahmet Riza Sahin.

**Visualization:** Tuba Turunc.

**Writing – original draft:** Tugba Arslan Gulen, Ebru Oruc.

**Writing – review & editing:** Tugba Arslan Gulen, Tuba Turunc, Ahmet Riza Sahin, Ebru Oruc, Mehmet Nurullah Kurutkan.
